# Experiences and lessons learned for programme improvement of micronutrient powders interventions

**DOI:** 10.1111/mcn.12496

**Published:** 2017-09-29

**Authors:** Marieke Vossenaar, Alison Tumilowicz, Alexis D'Agostino, Anabelle Bonvecchio, Ruben Grajeda, Cholpon Imanalieva, Laura Irizarry, Generose Mulokozi, Minarto Noto Sudardjo, Narantsetseg Tsevegsuren, Lynnette M Neufeld

**Affiliations:** ^1^ Independent Consultant Geneva Switzerland; ^2^ Global Alliance for Improved Nutrition Geneva Switzerland; ^3^ Strengthening Partnerships, Results, and Innovations in Nutrition Globally Arlington Virginia USA; ^4^ John Snow Inc. Arlington Virginia USA; ^5^ Instituto Nacional de Salud Publica Cuernavaca Mexico; ^6^ Pan‐American Health Organization Washington District of Columbia USA; ^7^ UNICEF Bishkek Kyrgyzstan; ^8^ Independent Consultant Lima Peru; ^9^ Tanzania Food and Nutrition Center Dar es Salaam Tanzania; ^10^ IMA World Health Dar es Salaam Tanzania; ^11^ Millennium Challenge Account Jakarta Indonesia; ^12^ World Vision International Ulaanbaatar Mongolia

**Keywords:** evidence‐based practice, infant and child nutrition, iron deficiency anaemia, micronutrients, monitoring and evaluation, programme evaluation

## Abstract

Continual course correction during implementation of nutrition programmes is critical to address factors that might limit coverage and potential for impact. Programme improvement requires rigorous scientific inquiry to identify and address implementation pathways and the factors that affect them. Under the auspices of “The Micronutrient Powders Consultation: Lessons Learned for Operational Guidance,” 3 working groups were formed to summarize experiences and lessons across countries regarding micronutrient powder (MNP) interventions for young children. This paper focuses on how MNP interventions undertook key elements of programme improvement, specifically, the use of programme theory, monitoring, process evaluation, and supportive supervision. Methods included a review of published and grey literature, interviews with key informants, and deliberations throughout the consultation process. We found that although much has been written and published about the use of monitoring and process evaluation to inform MNP interventions at small scale, there has been little formal documentation of lessons for the transition from pilot to scaled implementation. Supervision processes and experiences are not documented, and to our knowledge, there is no evidence of whether they have been effective to improve implementation. Improving the efficiency and effectiveness of interventions requires identification of critical indicators for detecting implementation challenges and drivers of impact, integration with existing programmes and systems, strengthened technical capacity, and financing for implementation of effective monitoring systems. Our understanding of programme improvement for MNP interventions is still incomplete, especially outside of the pilot stage, and we propose a set of implementation research questions that require further investigation.

## 1 INTRODUCTION

Strong evidence exists for the efficacy of many nutrition interventions, but the impact of these often falls short of expectations when integrated in programmatic contexts due to gaps in the quality of programme implementation (Bhutta et al., 2013; Habicht & Pelto, 2012; Leroy & Menon, 2008). Micronutrient powders (MNP), a mixture of vitamins and minerals, enclosed in single‐dose sachets, which are stirred into a child's portion of food immediately before consumption, are efficacious to reduce iron deficiency and anaemia in children 6–23 months of age (De‐Regil, Suchdev, Vist, Walleser, & Peña‐Rosas, 2013). However, results from programme evaluations in a number of countries have highlighted challenges in coverage, appropriate use, and adherence and show only modest biological impact (Rah et al., 2012).

There is increasing recognition in the field of nutrition that more and better information is needed to guide the design and delivery of programmes (Habicht & Pelto, 2014; Neufeld, Piwoz, & Vasta, 2016). Such information should be used for continual course correction to strengthen all aspects of implementation and revisit the design of programmes if implementation issues cannot be overcome. The generation of rigorous evidence to guide decision making for interventions has lagged in the nutrition community, and few studies have attempted to compile programmatic experience from multiple contexts. With at least 50 countries implementing MNP interventions across the globe, as of 2014, this lack of documented programme experience from MNP interventions has been highlighted as a “critical issue,” in the United Nations Children's Fund (UNICEF) NutriDash Global report (2015).

This paper is part of a series commissioned by the United States Agency for International Development (USAID) through the Strengthening Partnerships, Results, and Innovations in Nutrition Globally (SPRING) project to document experiences in planning, implementation, and monitoring of MNP interventions focused on young children and interpret implications for programmes globally. This paper examines MNP monitoring, process evaluation, and supportive supervision for continual programme improvement.

**Key messages**

- The ability to make evidence‐based decisions to improve micronutrient powders programme implementation is hindered by the lack of documented micronutrient powders experience, particularly for programmes implemented at scale.
- Monitoring and process evaluation more effectively inform programme improvements when based on programme theories operationalized through tools such as programme impact pathways and a shared learning agenda.
- Financial resources and technical capacity for monitoring and evaluation activities rely heavily on external institutions for support; support is often discontinued after the initial research stages of a programme.
- For effective programme improvement throughout the progression of the programme to scale, monitoring systems require prioritization of the information to be collected, and explicit feedback loops for viable data compilation, interpretation and utilization.
- Making evidence‐based programme improvement continual and effective requires close collaboration among micronutrient powders programme implementers and technical/research partners, careful planning, and adequate budget.
- A deeper understanding of factors that drive micronutrient powders coverage, utilization and impact, and at the same time are feasible to measure in resource constrained settings, is required to improve the efficiency and effectiveness of programme improvement activities.

## 2 METHODS

A consultative group consisting of 49 practitioners with knowledge in the implementation of MNP interventions was formed. The process is described in the executive summary of this series (Nyhus Dhillon et al., 2017). Briefly, under the auspices of “The Micronutrient Powders Consultation Lessons Learned for Operational Guidance,” three working groups (WGs) were established: planning and supply (WG1); delivery, social and behaviour change communication, and training (WG2); and monitoring, process evaluation, and supportive supervision for continual programme improvement (WG3). The focus of the consultation was to review interventions that were fairly well established and scaled, targeting children 6–23 months of age. However, as the consultative process unfolded, learnings from pilots and programmes with a wider target age (up to 59 months of age) were included, as well as some relevant lessons from emergency settings.

Each WG was charged with synthesizing available evidence from programmatic settings. The outcomes of this effort are presented in this paper for WG3 and elsewhere in this series for WG1 (Schauer et al., 2017) and WG2 (Reerink et al., 2017). WG3 consisted of 2 co‐chairs (LMN and AT) and 12 participants working for governmental institutions, multilateral and international organizations, universities, as well as independent consultants. WG members were based in Indonesia, Kyrgyzstan, Mexico, Mongolia, Peru, the Philippines, Switzerland, Tanzania, and the United States. WG3 participated in a yearlong (July 2015–July 2016) consultative process. It held two teleconferences to define the scope of the WG topic, participated in a meeting on October 19 and 20, 2015, in Washington, D.C., United States, exchanged emails, conducted key informant interviews, and reviewed literature.

The WG obtained primary data from key informants identified using purposive and snowball sampling (Table 1). Key informants either completed a questionnaire or were interviewed using the same structured questionnaire (Supporting Information S1). Follow‐up with key informants to confirm data and seek additional information was performed as necessary. WG members involved in implementation also completed questionnaires or were interviewed. Data were analysed by collating the information into a spreadsheet and identifying relevant information. We also identified key informants to provide information for case studies to take a more in‐depth look at context‐specific learning. Key informants provided expert opinion as part of their professional capacity and regular public health practice. Thus, the activities involved in the consultative process did not meet the human subjects research definition and were considered exempt by the John Snow, Inc. Institutional Review Board. Interview participants were told that their names would be confidential in all reports and manuscripts and that any information gathered would be summarized in manuscripts submitted for peer‐review publication.

**Table 1:** Characteristics of key informants^a^

Key informant number	Country(ies) of work^b^	Role of informant	Scale of Program^c^	Data collection method	Date of interview
1	Tanzania	Implementer	Subnational	Questionnaire	September 1, 2015
2	Mongolia	Implementer	National	Questionnaire	September 25, 2015
3	Indonesia	Policymaker	Pilot	Questionnaire	October 2, 2015
4	Kyrgyzstan	TA provider	National	Questionnaire Case study	October 10, 2015 March 18, 2016
5	Peru	Implementer	National	Questionnaire	October 12, 2015
6	Bolivia	Implementer	National	Interview	November 17, 2015
7	Mozambique	Policymaker	Pilot	Interview	January 14, 2016
8	Mexico	Implementer	National	Questionnaire	January 25, 2016
9	Kenya	TA provider	National^b^	Questionnaire	January 28, 2016
10	China	TA provider	Sub‐national	Questionnaire	March 7, 2016
11	Bangladesh	TA provider and implementer (joint interview)	National	Interview Case study	March 22, 2016 August 29, 2016
12	Guatemala	TA provider	National	Questionnaire	March 26, 2016
13	Kyrgyzstan	Two TA providers (joint interview)	National	Interview Case study	April 5, 2016 August 8, 2016
14	Bangladesh and Mexico	TA provider	National	Interview	August 3, 2016
15	Lao PDR	Implementer	Pilot	Interview	November 16, 2016
16	Madagascar	Implementer	Pilot	Interview	November 24, 2016
17	Rwanda, Uganda, Mozambique, Zambia, Cameroon, Namibia, Lao PDR, and Ethiopia	Two TA providers (joint interview)	Pilot	Interview	December 29, 2016

TA, technical assistance.

a Defined by the primary countries for which key informant provided experiences and learning.

b Defined by the stage of the intervention for which key informant provided experiences and learning.

c Interview focused on the experience of a national iron and folic acid supplementation programme, for comparison to the work done in micronutrient powders.

The WG obtained secondary data from a systematic search of peer‐reviewed and grey literature. The search inclusion criteria were implementation learning on MNP from inception through December 2015 and included a screening of abstracts, along with full texts when required, as described in more detail in the executive summary of this series (Nyhus Dhillon et al., 2017). A broad interpretation of relevance was applied when selecting literature to maximize the potential secondary data.

In this paper, we identify and summarize barriers and factors that facilitate adequate implementation of MNP interventions, highlighting examples from individual or multiple countries as appropriate. This analysis is not designed to provide results from any individual country. This analysis is also not designed to provide a detailed review of programme design, nor does it delve into specific monitoring indicators for programmes to use, which are covered elsewhere (HF‐TAG, 2013) and to a limited extent discussed in the delivery paper (Reerink et al., 2017). Rather, we focus on the existence and quality of systems for collecting, compiling, analysing, and using monitoring and research data for programme improvement. The findings from this review are presented as a series of statements that relate to current practice, followed by details of the findings from countries on which these statements are founded. Terms and working definitions for the content of this paper, defined based on literature and key informants, are presented in Box 1. The authors acknowledge that other definitions may apply outside the context of this paper.

**Box 1: Definitions of terms used in programmatic research by working group 3 (WG3): “Monitoring, Process Evaluation and Supportive Supervision for Continual Program Improvement”^a^**

**Efficacy:** demonstration that an intervention and/or a product “does more good than harm under optimum conditions” (Flay, 1986). Evidence of efficacy is demonstrated through randomized controlled trials, and is necessary but not sufficient for effectiveness.
**Effectiveness:** demonstration that an intervention and/or product “does more good than harm when delivered via a real‐world program” (Flay, 1986), i.e. whether it can produce the desired results when delivered in a programmatic context. Effectiveness is generated through program impact evaluation and given the diversity in logistical, administrative, political, social, and other factors across settings, results of impact evaluation in one setting may not be generalizable to others.
**Logic model:** a depiction (usually as table or figure) of the logical sequence and intended relationships between program inputs, activities, outputs, and outcomes (Kim et al., 2011; UNICEF, 2002).
**Logical framework (logframe):** transforms the logic model into specific indicators of process, output and outcome, intended to measure the progression and impact of the program as planned (Kim et al., 2011; UNICEF, 2002).
**Program impact pathway (PIP):** provides an explicit representation of the pathways by which the program (activities) achieve intended outcomes, taking into consideration non‐program factors (biological and/or contextual) that might facilitate or impede such impact (Kim et al., 2011; Rawat et al., 2013; Rossi et al., 2004).
**Monitoring:** continual tracking of inputs, activities and sometimes outputs of a program to assess performance against plans and identify areas for improvement. Monitoring may be internal (often referred to as routine monitoring), as part of continual systems, or external to the programs. For MNP programs, a detailed monitoring manual is available that lays out information to be collected and why, as well as alternatives of systems and processes to collect and utilize this information (HF‐TAG, 2013).
**Process evaluation:** looks inside the so‐called ‘black box’ of program implementation to see what happened in the program and how that could affect program outcomes or impacts (Saunders et al., 2005).
**Supervision:** review of documents and/or observing performance; often compared to check lists; standards of care, or other tools.
**Supportive supervision:** collaborative effort that involves discussion and joint problem‐solving, specifically designed to create opportunities to improve performance and gain confidence of workers (Also: mentoring).
**Quality improvement (QI):** systematic and continuous actions designed to lead to measurable improvement in program delivery and the health status of targeted groups. May utilize routine monitoring data and will include supportive supervision, but goes beyond with specific strategies to assess and improve performance through accreditation, performance‐based incentives, or others.
**Theory of change:** clear articulation of the intended impact of a program and the steps through which these impacts will be accomplished.

^a^MNP, micronutrient powders; PIP, Program impact pathway; QI, Quality improvement.

## 3 RESULTS

Sixty‐six peer‐reviewed articles, 16 guidance documents, and 45 programme reports or conference presentations with information on MNP programme implementation experiences were identified and reviewed (Nyhus Dhillon et al., 2017). Twenty‐one documents were identified as relevant for monitoring, evaluation, supervision, or programme improvement. Twenty key informants (KIs) were interviewed, completed questionnaires, or participated in the development of case studies (Table 1). Lessons from 22 countries in all six WHO geographic regions were included, some with multiple experiences with MNP pilots and programmes; case studies from Bangladesh (Box 2; KI 11) and Kyrgyzstan (Box 3; KIs 4 and 13) illustrate key experiences on the topics of this paper.

**Box 2: Bangladesh Case Study: Maternal, Infant and Young Child Nutrition (MIYCN) Home Fortification Program^a^^,^ b**

**Table:** 

Where	27 districts in rural areas (164 Sub‐districts) across the country and 6 urban slums in Dhaka District
When	The program started in July 2013 with current funding through 2018
MNP delivery strategy	BRAC's^c^ salesforce, female community health volunteers known as Shasthya Shabekas^d^ distribute MNP as part of IYCN promotion.
Target population	4 million children 6–59 months of age
Monitoring system	The monitoring system is financially supported by CIFF and was developed jointly by CIFF, GAIN, and BRAC, with on‐going data collection led by BRAC. Shasthya Shabekas and their supervisors complete monthly reports on supply, distribution, training, and social mobilization to promote good IYCF practices. Local reports are compiled and submitted on a monthly basis. These monitoring data are reviewed and findings fed back into the system to address identified challenges and improve performance.
A research and learning agenda for program improvement	In addition to the strong monitoring led by BRAC with technical inputs from GAIN, the program has benefited from a robust set of research activities, including in‐depth formative research, to understand barriers to improved IYCF practices and utilization of MNP. This includes coverage, utilization, barriers and opportunities for improvement, process and impact evaluation, and testing of alternative models as part of small implementation research studies. In an effort to consolidate all monitoring, research, and evaluation findings, and facilitate their interpretation and utilization for program improvement, GAIN with CIFF, BRAC, and icddr,b worked together to develop a learning agenda for the Bangladesh MIYCN Home Fortification Program Phase II. Continual update and discussion of the learning agenda permits triangulation of information across multiple sources, the consolidation of lessons learnt and their implications for program design and implementation and for emerging research priorities. It also serves as a repository to capture key messages and program modifications made as a result of those. It is a “living document” that all program partners routinely update with details of surveys or studies, key recommendations, and any program modifications that resulted from the research. The document is managed by GAIN and is in the process of developing a web‐based system soon, all partners are actively involved in keeping information up‐to‐date and in interpretation.

a CIFF, the Children Investment Fund Foundation; GAIN, Global Alliance for Improved Nutrition; icddr,b, International Centre for Diarrhoeal Disease Research, Bangladesh; IYCF, infant and young child feeding; IYCN, infant and young child nutrition; MIYCN, maternal, infant, and young child nutrition, MNP, micronutrient powders

b Based on information from key informant 11

c An international non‐governmental organization

d Frontline community health promoters.

**Box 3: Kyrgyzstan Case Study: Gulazyk^a^ Home Fortification Program^b^^,^ c**

**Table:** 

Where	Began as a pilot in Talas oblast (district) that later expanded to a national program (with the exception of Bishkek).
When	The pilot began in Talas in 2009. The program expanded to additional districts in 2010 and 2011, reaching national scale. Current supply problems have put the program on hold, however.
MNP delivery strategy	Trained health care providers distributed 30 sachets of MNP to eligible children every two months, free of charge. Communication was conducted through health care providers, village health community volunteers, and mass media campaigns.
Target population	Children 6–24 months of age. Reached around 250,000 children at national scale.
Monitoring system	Data collection by health care providers through health records. Supply data, coverage, and reasons for refusal were collected locally and then aggregated up the chain to the oblast‐level.
Process evaluation	Household surveys carried out by specially trained and hired survey staff, and health care personnel completed a short questionnaire. Indicators included product availability, coverage, adherence, KAP regarding MNP, receipt communications materials; knowledge and skills of medical workers, the quality of reporting documents.
How monitoring and process evaluation complement each other	The health system required rigorous recordkeeping for transparency and accountability purposes. This local data collection allowed decision makers to pinpoint where problems existed, something that surveys could not do. However, the records were not detailed enough to track the moving denominator (a result of children aging in/out of the program or migrating). Household surveys, on the other hand, were able to explore more in‐depth questions and provide representative estimates of complex indicators.
Transition from pilot to national scale	Both monitoring and process evaluation activities took place throughout the pilot and into the national scale‐up. Collecting similar data during the pilot allowed implementers and researchers to triangulate findings, and consistency between estimates indicated that internal monitoring systems were performing well. Process evaluation activities were slowly rolled back as the program scaled, with household surveys sampled using the Lot Quality Assurance Sampling method, instead of the more resource‐intensive proportional to size sampling of the pilot, before they ended with the study in 2013. The monitoring work continues, although supply difficulties have hampered its use in recent years.

a Local MNP product.

b KAP, knowledge, attitude, and practice; MNP, micronutrient powders

c Based on information from key informants 4 and 13.

### 3.1 A clearly articulated programme theory has been developed in many MNP interventions but has rarely been used throughout design and implementation to track progress and make course corrections

A common characteristic of MNP interventions considered to be effective is the clear mapping of programme theory, also known as a theory of change, through the use of a logic model. A logic model is essential to clearly articulate how activities will lead to impact. The monitoring manual for MNP interventions developed by HF‐TAG provides a generic logic model that can be adapted to specific country programmes (HF‐TAG, 2013). A programme theory operationalized in a logic model can bring stakeholders together around a shared understanding of how an intervention will achieve impact, as well as form the basis for solving problems that arise during implementation.

Although MNP implementers recognize the importance of a strong basis in programme theory, few authors document the use of programme theory and logic models, and their application to course correct during implementation. Loechl et al. (2009) explicitly used programme theory to guide the assessment of the feasibility and acceptability of distributing MNP through a food‐assisted maternal and child health and nutrition programme in Haiti. Ogunlade et al. (2011) used an implementation pathway to guide the process evaluation of MNP use in a preschool setting in South Africa, under normal field conditions and implementation constraints. Both studies relied on the use of a theoretical framework to link observed impacts and programme activities, providing useful information for programme replication or scale‐up. Results of this review suggest that most MNP programmes have developed or selected a guiding programme theory represented in a logic model, but these are commonly underutilized at critical stages of the programme. Although the reasons for not using a logic model are not clear and likely vary from context to context, part of the problem is lack of understanding or capacity within stakeholders to develop and effectively use it (KIs 4, 14, and 16). Key informants noted that logic models have not been used to develop comprehensive performance measurement or logical frameworks (also called logframes) with clearly defined and measureable indicators that reflect key inputs, activities, processes, and outputs of the programme and therefore have not been effective to identify and guide programme improvement (KIs 2, 4, 12, and 14). Moreover, the logic model often sits with programme evaluators or groups providing technical support, but are not owned and utilized by programme implementers (KIs 1, 14, 15, 16, and 17). Examples of logframes of key performance indicators are provided for the Bangladesh BRAC Maternal, Infant and Young Child Nutrition (MIYCN) Home Fortification Programme (Table 2) and the Kyrgyzstan Gulazyk Home Fortification Programme (Table 3).

**Table 2:** Logframe of key performance indicators for the Bangladesh BRAC Maternal, Infant and Young Child Nutrition Home Fortification Programme^a^

Programme components	Indicator	Source of information
Effective coverage	% of children 6–59 months of age who consumed at least 3 sachets in a week or at least 10 MNP sachets in last 30 days (1 month)	Coverage survey endline
	# of children covered divided by # of SS^b^ selling MNP in working areas	Routine monitoring
Practices improvement	% of caregivers who reports that the child does not like MNP due to side effects	Coverage survey endline
	% of caregivers who report appropriate IYCF practices (ICFI score)	Coverage survey endline
Programme intensity	% of HH who have received at least 1 visit by SS in the last 2 months	Coverage survey endline
MNP supplies	Proportion of SS who reports having insufficient supply of MNP to meet demand at any point during the reporting period	Routine monitoring
MNP sale	# of sachets sold by BRAC per year in working areas	Routine monitoring
Programme coverage	# of children age 6–59 months of age in target areas visited by SS and consumed at least 1 sachet	Routine monitoring
	# of children who have consumed 60 sachets over 6 months	Routine monitoring
SS performance	# of SS in Tier I divided by the total # of active SS	Routine monitoring
	# of SS in Tier II divided by the total # of active SS	Routine monitoring
	# of SS in Tier III divided by the total # of active SS	Routine monitoring
Programme roll‐out	# of subdistricts where the programme is implemented	Routine monitoring
	# of shasthya shebikas enrolled and trained in working areas	Routine monitoring
Enabling environment	National guidelines improved and inclusive of home fortification	Routine monitoring

a IYCF, infant and young child feeding; ICFI, infant and child feeding index; IYCN, infant and young child nutrition; MIYCN, maternal, infant, and young child nutrition; MNP, micronutrient powders; SS, shasthya shebikas.

b Frontline community health promoters.

**Table 3:** Logframe of key performance indicators for Kyrgyzstan Gulazyk^a^ Home Fortification Programme

Programme components	Indicator	Source of information
Biological impact	% of children 6–24 months of age in Talas oblast who have iron deficiency anaemia	External clinic‐based surveys
	% of children 6–24 months of age in Talas oblast who have other micronutrient deficiencies (vitamin A, folic acid deficiency)	External clinic‐based surveys
Adherence	% of children 6–24 months of age who consume at least the minimum acceptable dose of Gulazyk	External household monitoring survey
Coverage	% of children 6–24 months of age who received at least one package of 30 Gulazyk sachets	Health system administrative data; external household monitoring survey
	% of children 6–24 months of age who received a Gulazyk package/ration in the previous 2 months	Health system administrative data; external household monitoring survey
Availability/supply	% of the necessary supply received at each level of the primary health care system = amount of Gulazyk product received/amount needed	Health system administrative data
	% of health clinic distribution centres with Gulazyk in stock	External health centre monitoring survey
Quality of training	% of village health committee volunteers with adequate knowledge of Gulazyk	External monitoring survey of volunteers
	% of medical workers with adequate knowledge of Gulazyk	External health centre monitoring survey
	% of mothers with adequate knowledge of Gulazyk	External household monitoring survey
Training outputs	% of health promotion unit staff trained	Administrative records of health promotion units
	% of village health committee volunteers participating in the Gulazyk programme who were trained	Administrative records of health promotion units; external monitoring survey of volunteers
	% of health care providers who distribute Gulazyk that were trained	Administrative records of health care system; external monitoring survey of health care providers
Availability/supply	Adequate supply of educational materials = amount received/amount needed	Administrative records of health care system and health promotion units
Behaviour change activities	% of homes with a child 6–24 months of age who received at least one home visit from a village health committee volunteer	External household monitoring survey
	Number of radio broadcasts played on oblast radio station	Record/log of radio broadcasts
	% of mothers of children 6–24 months of age who heard at least one Gulazyk radio spot	External household monitoring survey

a Local micronutrient powder product.

Programme impact pathways (PIPs) go a step further than logic models by explicitly mapping the causal pathway of how an intervention results in biological impact (Neufeld et al., 2016) and the processes through which successful delivery of the intervention is expected to happen (Funnell & Rogers, 2011; Rawat et al., 2013; Rossi, Lipsey, & Freeman, 2004). PIPs account for contextual factors that might influence effectiveness and identify potential positive and negative unintended consequences (Bonvecchio et al., 2007; Kim, Habicht, Menon, & Stoltzfus, 2011; World Health Organization & UNICEF, 2008). They are particularly useful to understand factors that could facilitate or hamper intervention delivery beyond programme activities and specific corrective actions and/or complementary interventions that are essential to programmatic success (Kim et al., 2011). The importance of increasing their use to inform more programmatically relevant evaluations specifically for MNP interventions has been noted (Habicht & Pelto, 2012). Menon et al. (2013) used a PIP in a mixed‐methods process evaluation to highlight the role of demand creation in selling subsidized MNP by community health volunteers in rural Bangladesh. Looking beyond MNP interventions, IYCF programmes of Alive & Thrive in Bangladesh, Ethiopia, and Vietnam used PIPs to perform theory‐driven process evaluations to highlight successes of the project while identifying the need for programme improvements (Avula et al., 2013; Menon, Rawat, & Ruel, 2013; Rawat et al., 2013). Evaluation activities were led by a detailed PIP model, linking various data sources, relating evaluation with programme implementation timelines, and engaging with the programme implementation and management teams (Rawat et al., 2013). The PIP approach to guiding process evaluation was also used to inform nutrition and WASH interventions (Mbuya et al., 2015).

### 3.2 Monitoring systems that rely heavily on external support may not be viable when transitioned to scale

The structure of monitoring systems for MNP interventions varies substantially across the programmes reviewed. Monitoring systems predominantly depend on the type of delivery strategy (e.g., direct commercial sales; sales through community agents; free distribution through health sector; and emergency rations), scope and scale of the intervention, whether the programme includes research or specific learning activities, and if there are resources dedicated specifically to monitoring. When MNP are delivered through the health sector, monitoring is typically integrated into routine systems, but integration has been successful only where those systems are already functional.

Reasons to not integrate MNP monitoring into routine systems include external support or funding with limited duration and/or scale, as well as unstable supply of MNP. For example, in Mozambique, Ministry of Health (MOH) nurses distributing MNP as part of a pilot project used separate child registries specifically created for the purpose of the pilot. Data were compiled and analysed by MOH and the respective implementing partners—GAIN, the government's technical partner for their project in Sofala province, and Helen Keller International for Gaza province (KI 7). Similarly, during a pilot distribution of MNP in Nigeria through government‐implemented child health weeks, MNP distribution data were added to existing activity forms for the duration of the pilot but data were analysed by an external evaluator for the pilot (Korenromp et al., 2015). Although appropriate for small projects, the decision to separate data and its analysis may ultimately limit the speed and/or extent to which monitoring can be integrated into existing systems as the intervention expands.

When programmes start with a strong research focus, simplifying or scaling back MNP monitoring systems is typical when interventions scale‐up or are transitioned to local implementing agencies (KIs 13, 16, and 17). In Kenya, an effectiveness study of a social marketing and community‐based distribution programme had a robust system capable of tracking the complete delivery pathway of MNP, from supply through intake adherence through biweekly household surveys (Suchdev et al., 2010). After the study period when external financing and technical support from international partners was withdrawn, monitoring activities were scaled‐back substantially by the implementing non‐governmental organization. After research support ended, continued monitoring activities relied on reporting systems maintained by the implementer, rather than household‐level data. These continued activities focused on the supply and distribution of MNP and promotional materials, as well as promotional activities undertaken by distributors (Suchdev et al., 2013). Sale of subsidized MNP at small stores/kiosks and pharmacies in Tanzania had effective internal monitoring systems for tracking supply and sales when the partners were involved. Follow‐up monitoring systems implemented by a private distributor and the Government of Tanzania (through health facilities) declined after the withdrawal of donor support. Neither programme was able to integrate MNP monitoring into routine systems (KI 1).

Relying on private sector actors for distribution activities can complicate monitoring systems, as the distributors are not often accountable for reporting after the end of any pilot period (KIs 1, 14, and 15). This lack of engagement with programme staff, along with limited continuity with clients, makes reporting and monitoring of side effects, dropout, and intake adherence essentially non‐existent. This problem was partially overcome in Somaliland through the creation of a subgroup of 200 caregivers. Active follow‐up with these caregivers was done to inquire about any issues they were experiencing (e.g., side effects), and the information was used to modify the intervention. The Somaliland social marketing programme also created a free call‐in hotline where caregivers could access support and additional information on the MNP (PSI, n.d.).

### 3.3 Adequate allocation of financial and human resources for monitoring activities is essential but limited in many countries

In the most recent UNICEF NutriDash Global Report (2015), MNP programme implementers reported that monitoring was one of the top five challenges of intervention delivery due to limited capacity of personnel to monitor and analyse data, overall weak health systems, and limited resources for monitoring. An earlier UNICEF‐CDC global assessment of home fortification programmes similarly found that monitoring and evaluation was the top challenge, along with funding for programme delivery (Jefferds, Irizarry, Timmer, & Tripp, 2013).

Programme budgets often combine monitoring and evaluation into a single budget line (KI 14). Although related, the objectives and methodologies for monitoring and evaluation differ, and thus budget requirements for each vary. In many cases, it is difficult to disentangle the budget and ensure that each set of activities is adequately resourced. As discussed above, a comprehensive performance measurement framework that clearly articulates key indicators across the selected theory of change and identifies the data source and frequency for each indicator is critical for adequate budgeting. Experiences of national programmes suggest that monitoring systems are often more expansive and detailed when receiving external financial and technical support. After external support ends, the quality of monitoring systems tends to suffer (KI 14).

Our review reinforces these findings, identifying financial and human resources as important gaps in monitoring for MNP interventions in most countries examined. Key informants specifically highlighted the problem of insufficient resource allocation for monitoring, data collection, synthesis, dissemination, and utilization of quality information for programme decision making (KIs 1, 4, 6, 10, 13, 16). For example, common financial concerns raised in countries included lack of funds to hire additional personnel, incentivize the addition of monitoring functions to routine tasks, cover transportation costs for monitoring personnel, and provide adequate training of staff. Even in countries such as Bolivia, where MNP monitoring is integrated into mandatory, existing nationwide systems, human resources are often unavailable, leaving already overburdened national staff to deal with data gaps (KI 6).

Technical expertise was identified as critical for the design, quality and utilization of information in monitoring systems, as well as capacity for data analysis and interpretation, purposeful compilation, and feedback for effective programming. When technical assistance comes from organizations external to the implementing agency or country, there was a very strong call for capacity development so that local technical expertise is developed to manage programmes (KIs 4 and 17).

Interventions run through national health systems rely heavily on existing personnel. Limited capacity of health workers, in terms of skills and available time, and insufficient time and resource allocation for training and supervision, often due to heavy workload, were common concerns (KIs 1, 2, 4, 6, 11, and 17). Similarly, the additional burden to health workers imposed by add‐on interventions, such as MNP distribution, and the lack of clear and enforced accountability structures to ensure programme progress were identified as gaps (KIs 1, 2, 4, and 16). In Tanzania, for example, community health volunteers were expected to monitor consumption in addition to distribution (KI 1). The data on consumption monitoring were considered unreliable in this programme due to the complexity of the topic, the low capacity and inadequate training of the health workers, and the lack of systems to ensure accountability.

### 3.4 To be effective for programme improvement, monitoring systems require prioritization of the information to be collected, and explicit feedback loops for data compilation, interpretation, and utilization

The majority of MNP interventions include the collection of monitoring data, but even when adequately collected, key informants highlighted challenges to compile, interpret, and utilize results to inform decision making (KI 14 and 17). Similar to general observations related to monitoring capacity, key informants attributed the difficulty of effectively using monitoring data to limitations in capacity and technical support (KIs 1, 2, 4, 13, and 17), as detailed above. There is also a call for “real time” monitoring to encourage strategic use of data (KIs 5, 8, and 16). Key informants stress the importance of timeliness and the need for faster data turnover using innovative methods such as mobile technology and web‐based platforms to ease work burden and facilitate faster turnaround.

Creating a monitoring system that is effective and manageable in the context of programmes at scale with limited human, financial, and technological resources requires prioritization of a small set of indicators that are the main drivers of impact and benchmarks that trigger predefined actions to improve programme performance (KIs 2, 6, 11, 13, 16, and 17). Even where health information and monitoring systems are well established, the logistics for adding additional indicators relevant to MNP interventions need purposeful and cautious planning to ensure that critical information is gathered without overburdening the system. For example, in Madagascar, national monitoring staff were part of multiple revisions to forms before final indicators could be included in the system (KI 16). Programme sustainability concerns also result in additional barriers for MNP to overcome, as some countries will not incorporate indicators for interventions with uncertain futures into established monitoring systems. Key informants envision the ideal monitoring system as one that collects a few critical and reliable indicators, with minimum burden to well‐trained staff, and that allows immediate feedback and response (KIs 14 and 17). The Children's Investment Fund (CIFF) uses the “critical path” methodology (Coffin & Diaz Varela, 2014) to guide MNP programme improvement in Bangladesh. Similar to the PIP, the critical path seeks to map the pathway to impact then identify and closely track those steps likely to be the most critical to facilitate impact and prioritize the regular review of those indicators. Using the critical path methodology, CIFF identified a few essential indicators to illustrate progress towards targets; without improvement on these “milestone” indicators, impact is unlikely. The indicators prioritized in the logframe of the Bangladesh programme (Table 2) were chosen using the critical path methodology.

Furthermore, the programme in Bangladesh, in an effort to consolidate extensive research activities and facilitate their interpretation and timely utilization for programme improvement, a learning committee with a clear learning agenda was set up among partners. The learning agenda permits triangulation of information across multiple sources, the consolidation of lessons learned, implications for programme design and implementation, and for emerging research priorities (see Box 2 for description of learning agenda).

The programme in Bangladesh serves as an example of how a logical framework and monitoring data were used to solve problems faced during the implementation. Monthly monitoring data highlighted problems with MNP stock and supply, which was confirmed by quarterly stock‐in/stock‐out reports. GAIN delved deeply into the supply chain management system, triangulated all the available information from coverage survey findings, field visits' observation, and regular meetings with partners to decide course correction measures. These included practical actions such as renting additional warehouses at central and local levels, recruiting a supply chain manager, biweekly stock updates by partners, and prioritizing MNP supply in specific areas (KI 11).

### 3.5 Process evaluations are intended to complement monitoring systems and have been used in formative stages of a new programme, but more learning on how to improve implementation at scale is needed

Process evaluation activities are used for course correction of implementation (formative use) and to explain programme outcomes (summative use) (Habicht & Pelto, 2012; Saunders, Evans, & Joshi, 2005). Process evaluation is not intended to replace routine monitoring for timely feedback and corrective actions (Kim et al., 2015), as it generally requires extensive fieldwork, has a longer duration, and provides periodic versus continual feedback. Instead, process evaluation provides a limited‐duration opportunity for understanding the “how” of programme implementation in a way that allows for more meaningful discussion of changes needed to improve effectiveness.

We identified 15 peer‐reviewed papers in the published literature that included activities to investigate and describe factors (positive and negative) affecting programme implementation and ultimately programme impact (Table 4). The vast majority of published papers report on evaluations in the context of a pilot project (Creed‐Kanashiro, Bartolini, Abad, & Arevalo, 2015; Harris et al., 2012; Jefferds et al., 2015; Korenromp et al., 2015; Loechl et al., 2009; Mirkovic, Perrine, Subedi, Mebrahtu, Dahal, & Jefferds, 2016; Mirkovic, Perrine, Subedi, Mebrahtu, Dahal, Staatz, et al., 2015; Nguyen et al., 2016; Suchdev et al., 2010, 2013; Sun et al., 2011), with the exception of Bangladesh which reports on national programmes (Afsana, Haque, Sobhan, & Shahin, 2014; Angdembe, Choudhury, Haque, & Ahmed) and three large‐scale MNP distribution in refugee camps and in emergency contexts (de Pee et al., 2007; Kodish, Rah, Kraemer, de Pee, & Gittelsohn, 2011; Rah et al., 2012).

**Table 4:** Description of peer‐reviewed, published manuscripts explicitly documenting research on the process of implementing MNP programmes^a^^,^ b

Country/reference	Scale of programme at the time of evaluation, study year	Main study objective	Study design	Data sources
Bangladesh (Angdembe et al., 2015)	Implemented in 61 districts of the country at the time of study by BRAC, a national NGO (2012)	To assess adherence to MNP intake regime and associated factors in a community setting	Cross‐sectional study using quantitative methods	Interviews with caregiver using a semistructured questionnaire
Bangladesh (Afsana et al., 2014)	Implemented in 61 districts of the country at the time of study by BRAC, a national NGO (2013)	To describe BRAC's experience and achievements in scaling‐up a nationwide MNP programme	Mixed quantitative and qualitative methods	Periodic monitoring surveys, process evaluation survey, and rapid qualitative assessment
China (Sun et al., 2011)	Pilot [2 counties in Shan'xi province] (2008 & 2010)	To test the concept of public–private partnership to deliver MNP+ and to evaluate the effectiveness of marketing MNP+ through public–private partnership	Two cross‐sectional studies, convenience sample using quantitative methods	Cross‐sectional household surveys
Haiti (Loechl et al., 2009)	Pilot [Central Plateau region] (2005)	To assess the feasibility and acceptability of distributing MNP through a food‐assisted maternal and child health and nutrition programme using a programme theory framework in order to document programme processes	Mixed qualitative and quantitative methods, with design informed by clear programme theory	Structured observations, checks of beneficiary ration cards, exit interviews, focus group discussions, individual interviews, and survey data from the effectiveness evaluation
Indonesia (de Pee et al., 2007)	Emergency response (post‐tsunami; 2006)	To describe the post‐tsunami experience with distribution of MNP and to analyse the monitoring data gathered for the emergency response	Cross‐sectional, repeated surveys every 3–4 months using quantitative methods	Cross‐sectional household surveys
Kenya (Kodish et al., 2011)	Emergency response [Kakuma Refugee Camp] (2010)	To identify factors at the distal and proximal levels leading to the low uptake of MNP through a qualitative inquiry To understand perceptions of MNP and associated underlying causes of low uptake	Qualitative methods using an emergent design using an exploratory and iterative approach	Direct observations of food preparation and child feeding In‐depth interviews with community leaders, stakeholders, implementing partners, and beneficiaries Focus group discussions to examine perceptions and practices of beneficiaries
Kenya (Suchdev et al., 2010)	Pilot [Nyando District, Nyanza Province] (2007)	To describe monitoring of wholesale sales, household demand, promotional strategies, and perceived factors influencing MNP sales among vendors to improve ongoing programme delivery	Cluster‐randomized, longitudinal, cohort trial using quantitative and qualitative data	Cross‐sectional household surveys, sales records, biweekly household monitoring, vender focus groups, and key informant interviews
Kenya (Suchdev et al., 2013)	Pilot [Nyando District, Nyanza Province] (2007, 2008, 2009, 2010)	To evaluate the sustainability of subsidized MNP distribution by community‐based vendors after monitoring and marketing became the responsibility of the implementing organization when CDC funding for effectiveness study ended in 2009	Cross‐sectional, quantitative methods	Cross‐sectional household surveys Internal monitoring data collected by implementer
Kenya (Harris et al., 2012)	Pilot [Nyando District, Nyanza Province] (2007)	To evaluate the impact of “Safe Water and AIDS Project's” approach on equity of access to and use of health products (including MNP) and ultimately on health	Two‐year, longitudinal study and cross‐sectional surveys, quantitative methods	Household visits to monitor product purchases, product use, and household member morbidity; cross‐sectional household surveys
Kenya, Bangladesh, and Nepal (Rah et al., 2012)	Emergency response [refugee camps in one district each and 24 vulnerable districts in Bangladesh and Nepal] (2008–2010)	To describe the programme experience and findings of large‐scale MNP distribution in refugee camps and in an emergency contexts	Cross‐sectional and cohort panel data using quantitative methods	Cross‐sectional and panel data surveys
Nigeria (Korenromp et al., 2015)	Pilot [4 local government areas in Benue State] (2013, 2014)	To determine the feasibility of distributing MNP during biannual Maternal, Neonatal and Child Health Week events using a process evaluation framework	Cross‐sectional surveys, quantitative and qualitative methods	Surveys of caregivers and health workers, facility‐based observations of MNP distribution activities and cross‐sectional household surveys
Nepal (Mirkovic, Perrine, Subedi, Mebrahtu, Dahal, & Jefferds,, 2016)	Pilot [4 districts] (2011)	To identify modifiable predictors of intake adherence that could inform the design and implementation of MNP projects	Cross‐sectional using quantitative methods	Cross‐sectional household surveys
Nepal (Jefferds et al., 2015)	Pilot [4 districts] (2011)	To describe coverage of batches of MNP and factors influencing coverage for two MNP delivery models piloted in an integrated IYCF and MNP project	Cross‐sectional using quantitative methods	Survey among mothers and female community health volunteers
Nepal (Mirkovic, Perrine, Subedi, Mebrahtu, Dahal, Staatz, et al., 2015b)	Pilot [4 districts] (2011)	To examine the association between MNP consumption and select IYCF practices at 3 and 15 months after implementation of an integrated MNP/IYCF pilot programme in districts in Nepal	Cross‐sectional using quantitative methods	Cross‐sectional household survey
Peru (Creed‐Kanashiro et al., 2015)	Pilot [3 regions] (2010, 2011)	To explore and understand the acceptability and use of MNP among caregivers and health personnel in order to identify strategies to enhance its use by caregivers	Two‐phase qualitative study	In‐depth interviews and observations with caregivers and health personnel and home visits
Vietnam (Nguyen et al., 2016)	Pilot [four provinces: Thai Nguyen, Hai Phong, Quang Nam, and Ca Mau] (2014)	To describe pilot experiences with programme design, implementation, coverage results, and MNP use and compliance by caregivers and provide practical recommendations for programme scale‐up	Continuous monitoring and cross‐sectional surveys using quantitative and qualitative methods	Monitoring data (e.g., sales and distribution indicators); a qualitative survey with health workers; and a quantitative coverage survey with caregivers

a MNP, micronutrient powders; NGO, non‐governmental organization; AIDS, Acquired Immune Deficiency Syndrome; IYCF, infant and young child feeding.

b Presented in alphabetical order by country.

Programmes in the pilot phase tend to have a stronger research component including greater process evaluation with external surveys and more intense data collection, whereas full‐scale programmes tend to switch their focus to routine monitoring alone. In situations where a programme is well established and many of the key process questions have already been examined and answered, a routine monitoring system may be sufficient to ensure regular updates on progress. However, many implementers stated the value to continuing process evaluation beyond initial pilot stages or the beginning of a national programme, but the burden of data collection and analysis makes this difficult (KI 13).

An example of a programme with a strong research plan for the pilot stage is the MNP programme in Kyrgyzstan (Box 3), which included internal monitoring activities complemented by extensive external impact and process evaluations (Table 3; Lundeen, Imanalieva, Mamyrbaeva, & Timmer, 2013; Lundeen et al., 2010; Serdula et al., 2013). Process evaluation added to the data available for policymakers to decide whether and how to support the continued expansion of the intervention. In addition, it bypassed the limited statistical processing and analysis capabilities of the MOH, working with the National Statistics Committee to calculate complex indicators regarding coverage and adherence. After the programme reached national scale and process evaluation activities ended, implementers had to rely on internal monitoring indicators to inform decision‐making. After the pilot, implementers had to revise tools and simplify the data collection process to decrease the burden of this plan. This switch to routine monitoring has implications for the continuity of indicators and methodology of evaluation as the programme transitions from a small‐scale pilot into a full‐scale programme.

Key informants reported that it is often difficult to summarize and synthesize lessons of evaluation in the pilot phase and link these to decisions made at the moment of scale‐up due to insufficient transfer of technical expertise to the local level and lack of resources (KIs 1, 2, 4, 10, 13, and 17), as discussed above. Because of the lack of process evaluations on programmes at scale, there is less published about the factors that affect coverage, utilization, and impact among programmes at scale.

### 3.6 Process evaluations have required diverse methodologies and data sources to address the research questions most relevant to inform programme improvements

Exploring and testing hypotheses about the myriad of factors that affect how programme delivery happens and impact is achieved (or not) involves different types of research questions and subsequently different research methods and data sources. Among the 15 published papers in Table 4, seven relied on a mixture of quantitative and qualitative activities (Afsana et al., 2014; Korenromp et al., 2015; Loechl et al., 2009; Nguyen et al., 2016; Suchdev et al., 2010), or solely qualitative activities (Creed‐Kanashiro et al., 2015; Kodish et al., 2011). The vast majority of published papers relied on one or more surveys with caregivers for data collection; four used direct observations at the delivery stage or in the household (Creed‐Kanashiro et al., 2015; Kodish et al., 2011; Korenromp et al., 2015; Loechl et al., 2009), and three conducted in‐depth interviews with key informants or focus groups (Kodish et al., 2011; Loechl et al., 2009; Suchdev et al., 2010).

### 3.7 Supervisory systems and how they should be used to improve implementation is poorly documented for MNP interventions

Despite the general consensus among key informants that supervision for the purpose of quality improvement is important, we were unable to locate documentation related to supervision systems or processes for MNP interventions. Nor did process evaluations specifically address the issue of supervision and its role in improving programme implementation. Key informants reported that programmes infrequently collect data during supervisory visits and the potential role of supervision in programme improvement in general does not appear to be prioritized in MNP programmes (KIs 5, 11, 12, 16, and 17). Experiences with iron and folic acid supplementation in Kenya, however, suggest that this issue is not limited to MNP programmes. Supervisory visits in Kenya raised issues of unreliable iron and folic acid supply and addressed the problem through widespread advocacy. Despite this experience, stakeholders often assume that issues should be identified through quantitative monitoring systems and additional supervision is not necessary or prioritized (KI 9).

Similar to our finding on the sustainability of monitoring systems, supervision activities are also more intensive during the pilot phase of a project and those with research components and are often not sustained during scale‐up. For example, although active supervision visits were part of the pilot in Madagascar, there was insufficient budget to meet all the costs for the planned activities and supervision efforts were ultimately scaled‐back to focus on communities struggling with distribution (KI 16). In Lao PDR, supportive supervision efforts were regular but informal, relying on verbal feedback to supervisees as the main form of capacity building (KI 15). Guatemala integrated MNP supervision into an existing system that followed formalized checklists and relied on review of previous supervision data in the following visit (KI 12). However, these regular visits prove just as burdensome for health workers as many of the data collection tasks that have been placed on them.

## 4 DISCUSSION

Regular monitoring, process evaluation, and supportive supervision should be essential components of any MNP intervention —and nutrition programmes more broadly. This review and consultation identified factors that facilitate or impede the ability to make evidence‐based decisions to improve MNP programme implementation, starting with the awareness of the importance of such measures by stakeholders and decision makers. The findings presented here are similar to those of a recent review of government information systems to monitor complementary feeding programmes for young children, which concludes that all programmes need internal monitoring to implement effective programmes. The review highlights the use of programme description and conceptual models to develop a monitoring system, including indicators and tools, that is feasible to implement and maintain throughout the programme and can be used for programme improvement. Complementary feeding indicators should fit their context and provide data that can be part of a decision making and programme improvement process (Jefferds, 2017).

Literature and programme experiences illustrate the importance of identifying and addressing implementation challenges to realize the potential impact of MNP interventions, but little has been written to compile the experience of how such monitoring and evaluation systems have worked. Although we aimed to systematically gather available published literature, and to find key informants in countries where MNP were implemented, this consultative process had various methodological limitations. First, outside of the realm of research studies, information regarding routine monitoring data are often not available. Most publications were focused on efficacy and lacked an explicit focus on programme learning; therefore, many of the documents were not informative regarding processes for programme improvement. The lack of action‐oriented research in the area of nutrition is acknowledged more generally (Pham & Pelletier, 2015).

Second, this paper does not reflect all monitoring systems, because it relies on cases where published literature discussed the system or the authors were able to interview someone familiar with it. Many stakeholders are involved in MNP implementation over the course of a number of years. Due to time constraints and the retrospective nature of the programme, it was not possible to speak with representatives from all stakeholder groups. When possible, the authors attempted to verify details with information from another data source. Although many countries and international organizations were included in this process, the authors of these papers acknowledge that some countries and experiences that may have added to the learning were not included. This type of summative process on programme experiences is still fairly methodologically new (Green, 2008) and subject to a certain amount of subjectivity. The subjectivity in our approach includes our snowball sampling, the use of expert opinion as the primary data and subject to author interpretation and biases. The lack of published or documented experience, particularly for at‐scale programmes and supportive supervision, means that the results presented here are indicative of the situation but cannot be considered a definitive or exhaustive review of the issues.

To date, most learning on MNP interventions has been from programmes implemented at small scale with a high level of external technical assistance, financing, and process evaluations during the start‐up phase. The extent to which the evidence generated from these evaluations has informed subsequent changes in programme design and/or implementation, however, is often not adequately captured in programme reports or publications. Similarly, the high level of external support during start‐up phases has not necessarily translated into strong monitoring or ongoing evaluation as programmes transition into routine monitoring systems. Most platforms used for MNP delivery have limited human and financial resources to sustainably adopt new indicators, collect and analyse data, and report results. Successful integration of evidence‐based programme improvement activities into routine systems requires technical support (at a minimum at the programme initiation stage), careful planning, and adequate budgeting. Capacity assessment and development (if needed) should be included during that phase to ensure systems can continue to inform programmes and evolve once external support is reduced.

Understanding the processes through which programmes achieve high acceptance of MNP and high adherence to recommendations regarding the use of MNP is critical to informing programme improvement, replication, and scale‐up. Yet although programme theory frameworks, such as logic models and PIPs, are considered necessary, the randomized control trials dominating the MNP literature are rarely designed to pinpoint implementation constraints. As a result, the biological pathway to impact for MNP is well articulated but programme implementers have insufficient knowledge of the processes through which successful delivery of the intervention could be expected to happen. This observation echoes other recent calls to improve the capacity of nutrition researchers to measure intermediary steps between programme inputs and biological outcomes and assess implementation fidelity (Habicht & Pelto, 2012). Programme implementers need this information to identify the critical path to impact, prioritizing what information is necessary and sufficient to inform subsequent action.

Improving the efficiency and effectiveness of programme improvement activities including monitoring, process evaluation, and supportive supervision requires identification of a small set of indicators critical for detecting implementation challenges, integration with existing programmes and systems, strengthening technical capacity, and financing for its implementation. More implementation research on programmes at scale and greater efforts to document and share research results and programme experiences is also needed.

Areas of needed implementation research identified during the consultative process include the following:

- Examine how to effectively link monitoring and process evaluation to decision‐making processes;
- Document lessons in how to sustain monitoring systems from pilot to larger scale;
- Assess how to manage monitoring across multiple, integrated interventions;
- Document how to carry out effective supportive supervision, especially in contexts with high turnover of MNP staff; and
- Determine how to link MNP interventions to broader health systems, strengthening activities at all levels.

## CONFLICTS OF INTEREST

The authors declare no conflicts of interest.

## CONTRIBUTIONS

This paper was written by MV, AT, LMN with substantial contributions from AD, ABA, RG, CI, LI, GM, NMS, and NT. AT and LMN chaired the working group, and AD served as the secretariat. All authors were involved in developing the paper concept and have reviewed and approved the submitted manuscript.

## Supporting information

Supporting Information S1. Supplementary Material 1
Working Group 3 Questionnaire on monitoring, process evaluation, and supportive supervision for continual program improvement (as provided to key informants and/or used as interview guides)
