# Supplementary material for: Experiences and lessons learned for programme improvement of micronutrient powders interventions
Source: Matern Child Nutr. 2017 Sep 29;13(Suppl 1):e12496. doi: 10.1111/mcn.12496 (PMC5656835; doi:10.1111/mcn.12496)
Supplement: Supplementary file 1 — Supporting Information S1. Supplementary Material 1 Working Group 3 Questionnaire on monitoring, process evaluation, and supportive supervision for continual program improvement (as provided to key informants and/or used as interview guides) [file MCN-13-e12496-s001.doc]

**Supplementary Material 1**

**Working Group 3 Questionnaire on monitoring, process evaluation, and supportive supervision for continual program improvement (as provided to key informants and/or used as interview guides)**

| **Questionnaire: Monitoring, Process Evaluation, and Supportive Supervision for Continual Program Improvement Working Group** |
| --- |
| 1. In what country(ies) do you have experience working with MNP programs? 2. Please indicate your role in the program and length of involvement. 3. Please complete the following:  - Country/region: - Scale: *Pilot/national (please indicate year started)* - Target population and coverage: *Age, region, SES, etc.; Number of children reached* - Delivery model: - Implementing partners - Program funding and duration: - M&E funding and duration: *If possible, please provide budget for M&E as an absolute value or % of total budget.* *Are there separate budgets for monitoring and for evaluation?* - Government engagement: *National policies that support MNP program* |
| **Internal/Routine Monitoring**   - Definition of routine monitoring: continual tracking of input, activities, sometimes outputs internal to the implementer; often tracked against objectives or performance standards. |
| ***Set-up and function*** |
| 1. What institutions are responsible for the internal monitoring system? Including the roles of the different institutions. |
| 1. Describe the internal monitoring system and whether it is integrated with other monitoring systems (e.g. through existing government Monitoring Information Systems (MIS)) and/or independent. |
| 1. Has there been a log frame or similar tool developed for the program? Is there a performance measurement framework linked with that? Has this been used in any way to inform monitoring activities? |
| 1. Could you share with us any documentation of the internal monitoring system? For example, logical framework, logframe, forms. |
| ***Monitoring indicators*** |
| 1. In your opinion and for your needs, what are the most important indicators/priorities for monitoring? |
| 1. Has the program monitored indicators at the level of the caregiver/child, such as acceptability or adherence? If so, please describe the experience doing so. |
| ***Human resource requirements*** |
| 1. Could you describe the human resources involved in carrying out the monitoring system at the level of data collection, data analysis and data interpretation? |
| 1. How were people trained to conduct internal monitoring? |
| 1. Describe the process of analyzing monitoring information and using results for course correction, including roles of the different institutions involved, frequency of reporting and/or course correction. |
| ***Challenges and thoughts about how the system could be improved*** |
| 1. What are challenges that are faced in conducting internal monitoring? |
| 1. Could you describe differences between the planned internal monitoring system and the reality in the field? |
| 1. What would your ideal internal monitoring system look like? |
| 1. Other thoughts for our group? |
| **External Monitoring/Evaluation** |
| ***Purpose*** |
| 1. Does the program have external monitoring? If so, what are the main goals of the evaluation? |
| ***Set-up and function*** |
| 1. What institutions are responsible for the external monitoring system? Including the roles of the different institutions. |
| 1. Describe the external monitoring system and frequency. |
| 1. Could you describe any key decisions that had to be made about the external monitoring system and how the decisions were made? |
| 1. Could you share with us any documentation of the external monitoring system? For example, logical framework, logframe, forms. |
| ***Monitoring indicators*** |
| 1. In your opinion and for your needs, what are the most important indicators/priorities for external monitoring? |
| 1. Has the program monitored indicators at the level of the caregiver/child, such as acceptability or adherence? If so, please describe the experience doing so. |
| ***Human resource requirements*** |
| 1. Could you describe the human resources involved in carrying out the monitoring system at the level of data collection, data analysis and data interpretation? |
| 1. How were people trained to conduct external monitoring? |
| 1. Describe the process of analyzing monitoring information and using results for course correction, including roles of the different institutions involved, frequency of reporting and/or course correction. |
| ***Challenges and thoughts about how the system could be improved*** |
| 1. What are challenges that are faced in conducting external monitoring? |
| 1. Could you describe differences between the planned external monitoring system and the reality in the field? |
| 1. What would your ideal external monitoring system look like? |
| 1. Other thoughts for our group? |
| **Process Evaluation – Use In Course Correction Measures**   - Process evaluation: collection of information to assess the quality of implementation using in comparison to planned processes. May use studies, surveys, routine monitoring data or combinations. |
| 1. How reliable is the data that is received through internal monitoring? |
| 1. How has internal monitoring information been used? |
| 1. Has the internal monitoring data been used for program improvement? If so, please describe processes and responsibilities. |
| 1. How reliable is the data that is received through external monitoring? |
| 1. How has external monitoring information been used? |
| 1. Has the external monitoring data been used for program improvement? If so, please describe processes and responsibilities. |
| **Quality Improvement** |
| ***Description of experience*** |
| 1. Has there been a continuous program improvement or quality improvement process? If so, using what methods? |
| 1. Have the following systems been set up? If so, please describe the activities.  - Forming a quality improvement team - Setting aims and measurements for improving program performance - Developing tools (e.g. quality assurance checklists) - Operational research aimed at improving program performance |
| 1. To what extent is that process directly linked with the routine monitoring, external monitoring and/or supervision systems? |
| **Supervision**   - Definition of supervision: Review of documents and/or observing performance; often compared to check lists; standards of care, or other tools - Definition of supportive supervision: collaborative effort that involves discussion and joint problem-solving, specifically designed to create opportunities to improve performance and gain confidence of workers (Also: mentoring) |
| ***Purpose*** |
| 1. Describe the main purpose of supervision in the context of this program (evaluation of performance, build capacity of supervisees, monitoring) |
| ***Structure*** |
| 1. Describe the supervisory roles of the institutions involved in the program. |
| 1. Describe the supervision structure of the program (e.g. from bottom to top) |
| ***Training/capacity building/tools*** |
| 1. How were supervisors trained to do supervision? |
| 1. Are there tools that were developed for supervision visits? Could you share them with us? And describe the experience using the tools? |
| 1. As now, are there any specific linkages between the information that is collected as part of routine monitoring and the supervision activities that you have described? If so, please describe how this works. |
| ***How has supervision been used?*** |
| 1. In your opinion and for your needs, what are the most important priorities for supervision? |
| 1. How would you describe the usefulness of the supervision that has happened as part of the program? |
| ***Challenges and thoughts about how supervision could be improved*** |
| 1. What are challenges that are faced in conducting supervision? |
| 1. How are ways that you see supervision could be improved and/or made more useful? |
| 1. Have supervision activities influenced program performance? If so, please describe. |
| 1. Other thoughts for our group? |
| **Sustainability and Scale** |
| ***Description of experience*** |
| 1. Describe the scale of your program (national, regional, pilot, etc). Please include year. |
| 1. Describe how the program in currently envisioned in terms of sustainability- has there been specific discussions related to the duration of the program? |
| 1. What are the funding sources in the short, medium, and longer term? |
| 1. How would you characterize the sustainability of this program? Any issues that you consider would affect its sustainability? |
| 1. In your opinion, what are key factors to ensure sustainability of MNP programs? |
| ***Evolution of the scale-up*** |
| 1. What processes were put in place to plan for scale, such as allocating budget, getting commitment and buy-in among many other relevant issues? |
| 1. Did the project start with a pilot? If so, was there a specific planning exercise to go from pilot (or small scale (to scale up)? If so, describe this and any tools used to support it. |
| 1. Describe how the program evolved to be large scale (if applicable) |
| 1. Describe the change in monitoring systems as program evolved to be large scale (if applicable) |
| ***Funding*** |
| 1. How is the program funded? Add funders and time frame. |
| 1. Are multiple sources of funding included? If so, describe. What would be the implications if any one of those funding sources were to be withdrawn for project sustainability? |
| 1. If donor support were to be withdrawn, is the government or private sector ready/prepared to take on MNP distribution? If not, what do you think needs to happen for this? Is this feasible in the given context? |
| 1. How is the monitoring and evaluation of the program funded? Add funders and time frame. |
| 1. How much funds are available for monitoring and evaluation (as % of program). Is there a separate budget for monitoring versus evaluation, if so specify. |
| ***Success and challenges related to scale-up*** |
| 1. Describe the successes that have happened in the process of scaling-up this program. What have been the main determinants of these successes? |
| 1. Describe the challenges that have happened in the process of scaling-up. What were the causes of those challenges? |
| 1. What will be the opportunities and/or challenges for maintaining the program at scale in the future? |
